# Experimental Evaluation of Maximum-Likelihood-Based Data Preconditioning for DE-SPECT: A Clinical SPECT System Constructed With CZT Imaging Detectors

**DOI:** 10.1109/trpms.2024.3520668

**Published:** 2024-12-24

**Authors:** Yifei Jin, E. M. Zannoni, Ling-Jian Meng

**Affiliations:** Department of Nuclear, Plasma and Radiological Engineering, University of Illinois at Urbana–Champaign, Champaign, IL 61801 USA; Department of Nuclear, Plasma and Radiological Engineering, University of Illinois at Urbana–Champaign, Champaign, IL 61801 USA. She is now with the Walker Department of Mechanical Engineering, The University of Texas at Austin, Austin, TX 78712 USA; Department of Nuclear, Plasma and Radiological Engineering, and the Department of Bioengineering and Beckman Institute for Advanced Science and Technology, University of Illinois at Urbana–Champaign, Champaign, IL 61801 USA

**Keywords:** Cadmium zinc telluride (CZT) detectors, maximum-likelihood estimation, spatial distortion, single photon emission CT (SPECT)

## Abstract

This study introduces a novel maximum-likelihood-based data preconditioning method for a 3-D position sensitive cadmium zinc telluride (CZT) detector used in the dynamic extremity-single photon emission computed tomography imaging system, an organ-dedicated Single-Photon Emission computed tomography system optimized for imaging peripheral vascular diseases in lower extremities. The 3-D CZT detectors offer subpixel resolution of ~0.5 mm FWHM in X-Y-Z directions and an ultrahigh energy resolution of 3 keV at 200 keV, 4.5 keV at 450 keV, and 5.4 keV at 511 keV. Given the intrinsic challenges posed by pixel boundary issues, spatial distortions, and nonuniformity inherent in large-volume, high-resolution CZT detectors, we proposed a Maximum-Likelihood-based preconditioning technique to reconstruct the projection, which effectively mitigates the pixel boundary issue and deconvolves the distortions and nonuniformity in detector responses. To facilitate the preconditioning step, we used sheet-beam scanning to measure the distortion map of the CZT detectors. We have evaluated our data preconditioning technique through extensive experimental evaluations, including Tc-99m sheet-beam scanning and image reconstruction of an image quality phantom. These results not only demonstrated the efficacy of the technique in reducing the impact of pixel boundary issues and correcting for spatial distortions. The proposed data preconditioning technique could potentially be applied across various types of imaging sensors.

## I. Introduction

Room temperature semiconductors, such as cadmium zinc telluride (CZT) are gaining widespread interest in many applications, such as astronomy, homeland security and medical imaging, notably for their exceptional energy, and spatial resolution. In medical imaging, there are increasing interests in photon-counting computed tomography (CT) [1], [2] and single photon emission CT (SPECT) [3], [4], [5], improving diagnostic capabilities with enhanced image quality (IQ). The dynamic extremity SPECT (DE-SPECT) is a high-performance SPECT system currently under construction [6]. It utilizes 48 large-volume, high-resolution 3-D position-sensitive CZT detectors. These detectors are capable of detecting multiple interactions induced by a single incident gamma-ray with a subpixel resolution of ~0.5 mm in 3 dimensions and an ultrahigh energy resolution of 3 keV at 200 keV, 4.5 keV at 450 keV for each detected interaction [7].

However, nonuniformity and defects in CZT crystals can lead to inaccuracies in positioning gamma-ray interactions. Furthermore, imperfections in the practical design of the pixels and electronics, as well as subpixel positioning algorithms could contribute to systematic errors in event positioning, especially for interactions that occurred close to pixel boundaries. These event positioning errors will lead to artifacts in projection and compromise the quality of reconstructed images. Consequently, implementing data preconditioning becomes a critical step before image reconstruction to address these issues and ensure the quality of the resultant images.

Spatial distortion in gamma-ray imaging systems has been a subject of ongoing research, with methodologies evolving over time [8], [9], [10], [11]. Early methods, like the centroid calculation by Kinahan and Karp for NaI (Tl) detectors [12], and Johnson et al.’s flood map approach for gamma camera uniformity [13], set the stage for further innovations in Anger cameras [14], [15], [16], [17]. Recent developments have focused on position-sensitive avalanche photodiodes, leveraging advanced techniques, such as Fourier analysis and maximum-likelihood estimations for improving the precision of event positioning [18], [19], [20]. These efforts underlie a shift toward more sophisticated computational methods to refine gamma-ray detector accuracy and efficiency in various applications. Despite these efforts, the intrinsically different physical properties of scintillator-based detectors and semiconductors make them less inspiring in solving the issues in CZT crystals.

In recent years, there have been some computation-based methods that were explored for improving event positioning in scintillation and semiconductor imaging detectors. Barrett et al. [21] explored maximum-likelihood methods specifically designed for gamma-ray detectors, aiming to optimize the signal processing for these systems. España et al. [22] introduced a novel calibration method for SPECT monolithic scintillation detectors. Their technique utilized un-collimated sources and a straightforward geometry to achieve fast calibration. Li and Furenlid [23] also presented a fast calibration method with sheet beam scanning for monolithic detectors. Jeon et al. [24] proposed a distortion correction method that employed tomography transformations, specifically focusing on position error correction in gamma-ray imaging detection systems. The growing interest in incorporating machine learning in gamma-ray imaging reflects an evolving trend. Various deep learning techniques have been developed to estimate gamma ray interaction locations in monolithic scintillation crystal detectors [25], [26]. In addition, Yang et al. [27] have created a fully connected neural network that jointly estimates interaction positions and energy deposition in CdTe/CZT imaging sensors. Their approaches showcased the potential of machine learning in achieving high accuracy while reducing computational costs. However, some artifacts are generated by the network which might be not suitable for SPECT imaging. While these methods do not directly address issues in CZT detectors, they offer valuable insight into potential solutions.

Although CdTe/CZT detectors have gained increasing interest over the past few decades, research specifically focused on spatial correction within these detectors remains limited. One notable contribution in this area is by Kim et al. [28], who presented a flattening method for error correction in virtual Frisch-grid CZT detectors.

Existing methods in this area developed for pixelated and monolithic scintillators are not fully compatible with semiconductor detectors. The primary reason is the fundamental difference in their operation principles. Moreover, the lack of intermediate signals in commercial semiconductor detectors like those used in the DE-SPECT system further complicates the implementation of these traditional methods. This study aims to fill this gap by introducing a maximum-likelihood-based data preconditioning technique to address several issues associated with event positioning processes. Unlike most existing techniques that require access to intermediate signals, our proposed technique only utilizes final spectral and spatial information for correction. This makes it widely applicable to other position-sensitive detectors as well.

The primary objective of this study is to propose and experimentally evaluate a maximum-likelihood-based data preconditioning technique, which is applicable to CZT detectors and potentially other detectors. The content of this paper is organized as follows. First, we introduce the maximum-likelihood-based data preconditioning technique and detail the theoretical foundation of this technique. Second, we describe a sheet-beam scanning method employed to obtain the detector response function (DRF), a crucial component in the data preconditioning process. Third, we discuss the preprocessing and postprocessing steps implemented to enhance projection quality. Finally, we assembled a pinhole imaging system using a single CZT detector and conducted Tc-99m phantom studies in both projection and image domains. These studies are designed to demonstrate the practical effectiveness of our proposed preconditioning technique in both projection and imaging domains, thereby highlighting its potential application in the field of medical imaging.

## II. Material and Methods

### A. 3-D CZT Imaging Spectrometer

The DE-SPECT imaging system is constructed with 48 3-D position-sensitive CZT detectors of an 1-cm-thickness [6]. Each CZT detector consists of 2 × 2 CZT modules. Each module is 2.2 × 2.2 × 1.0 cm^3^ in size and has 11 × 11 anode pixels and a continuous cathode. Each pixel metal contact is 1.84 mm in size with a pitch of 1.9 mm. It provides an ultrahigh energy resolution (3 keV at 200 keV, 4.5 keV at 450 keV, and 5.4 keV at 511 keV) [7]. This high resolution facilitates multifunctional imaging capabilities of the DE-SPECT system.

Although the anode pixel pitch is 1.9 mm, it can offer a subpixel resolution of ~0.5 mm FWHM in x- and y-directions with subpixel positioning [7]. The principle is as described below. When a gamma-ray interaction occurs, the induced signal on the pixel collecting the electron clouds will rise to an amplitude. Neighboring noncollecting pixels also detect transient signals due to weighting-potential crosstalk among pixels. These transient signals initially rise as the electron cloud travels through the detector bulk and then fall to zero when the signal electrons reach the collecting anode pixel(s). The peak amplitudes of these transient signals induced on the noncollecting pixels are sensitive to the x- and y-positions of the electron cloud and can be used to derive the position of the interaction at a spatial resolution smaller than the pixel pitch size (1.9 mm). The CZT detector can also provide DOI information at ~0.5 mm FWHM resolution, which is determined by cathode-to-anode ratio (CAR) and measuring the charge-drifting time inside the detector [29], [30], [31].

**Fig. 1.**
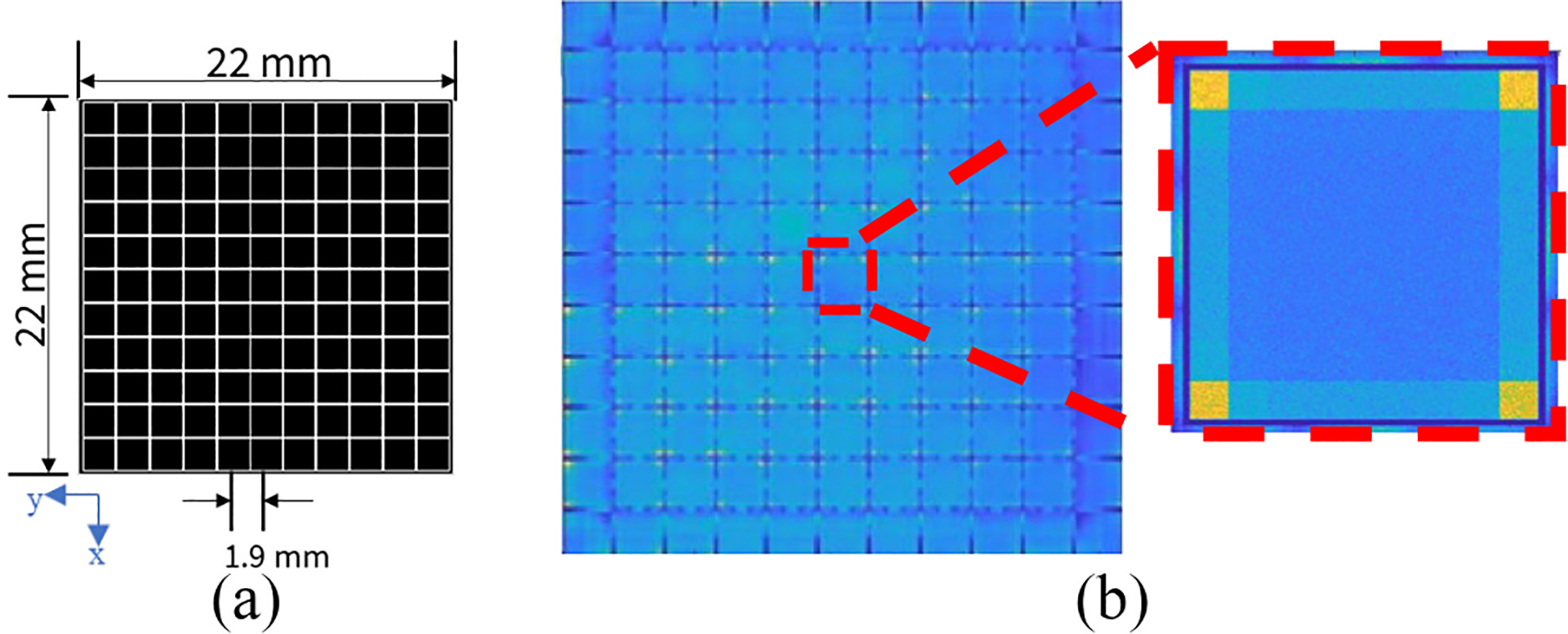
(a) Anode structure of one CZT module. (b) Experimental projection of abnormal pixel boundary acquired from a Co-57 flood source (energy window: [120 keV, 124 keV]).

However, when calculating subpixel positions, issues arise if the interaction occurs close to the pixel boundary. In such cases, the detected signals do not provide sufficient information to differentiate true charge-sharing events from noncharge-sharing events that induce signals on adjacent noncollecting pixels due to weighting potential crosstalk. This would inevitably lead to some events that are positioned incorrectly as shown in Fig. 1. Even with a uniform flood irradiation, some events are clustered near the pixel boundaries, creating the square-grid pattern at a pitch size of 1.9 mm.

**Fig. 2.**
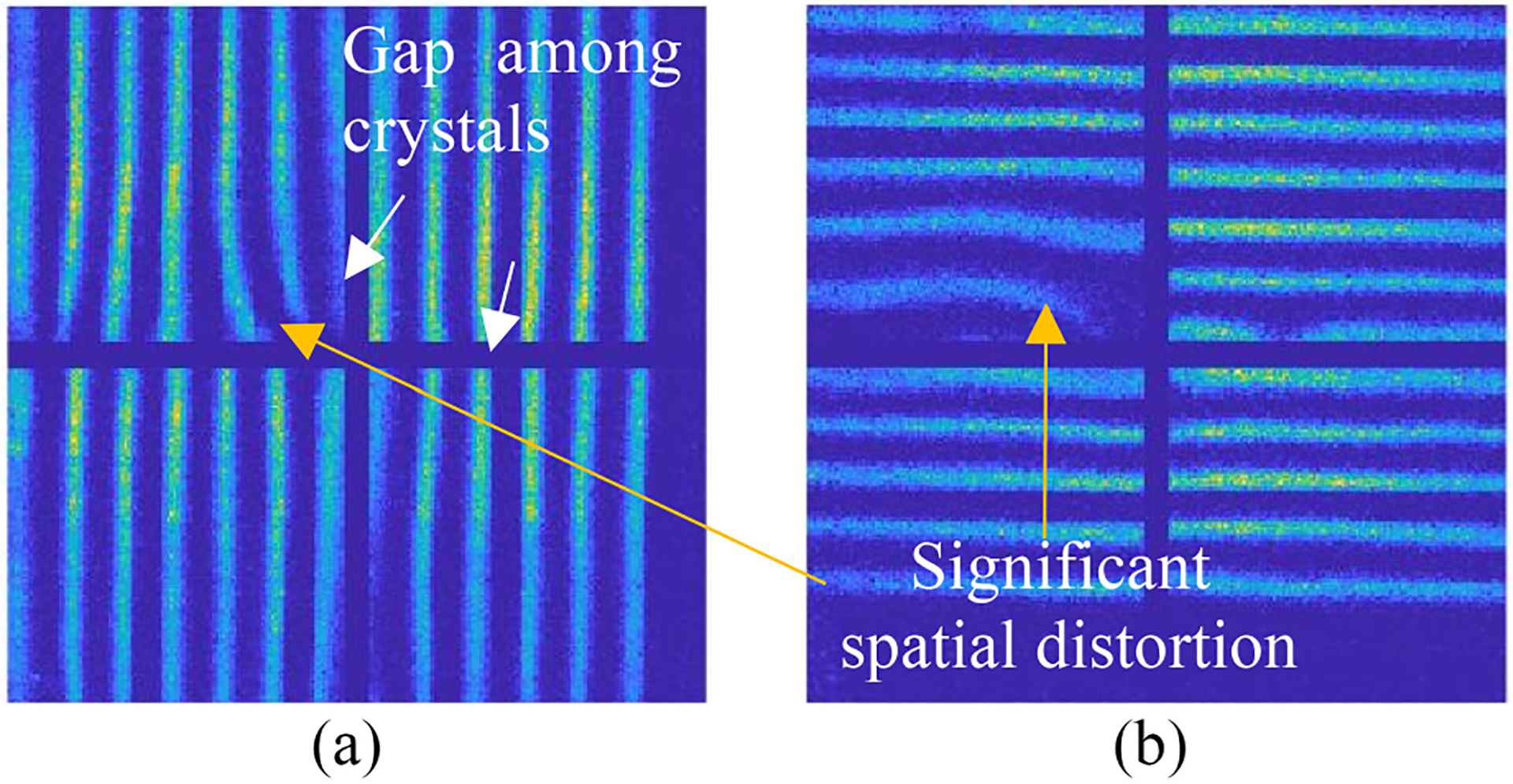
Experimental projections synthesized from Co-57 sheet beam scanning in 2 directions. The step size is 3 mm. Gap among crystals and spatial distortions are highlighted in the figure. The serial number of the detector is M-00–01-070.

**Fig. 3.**
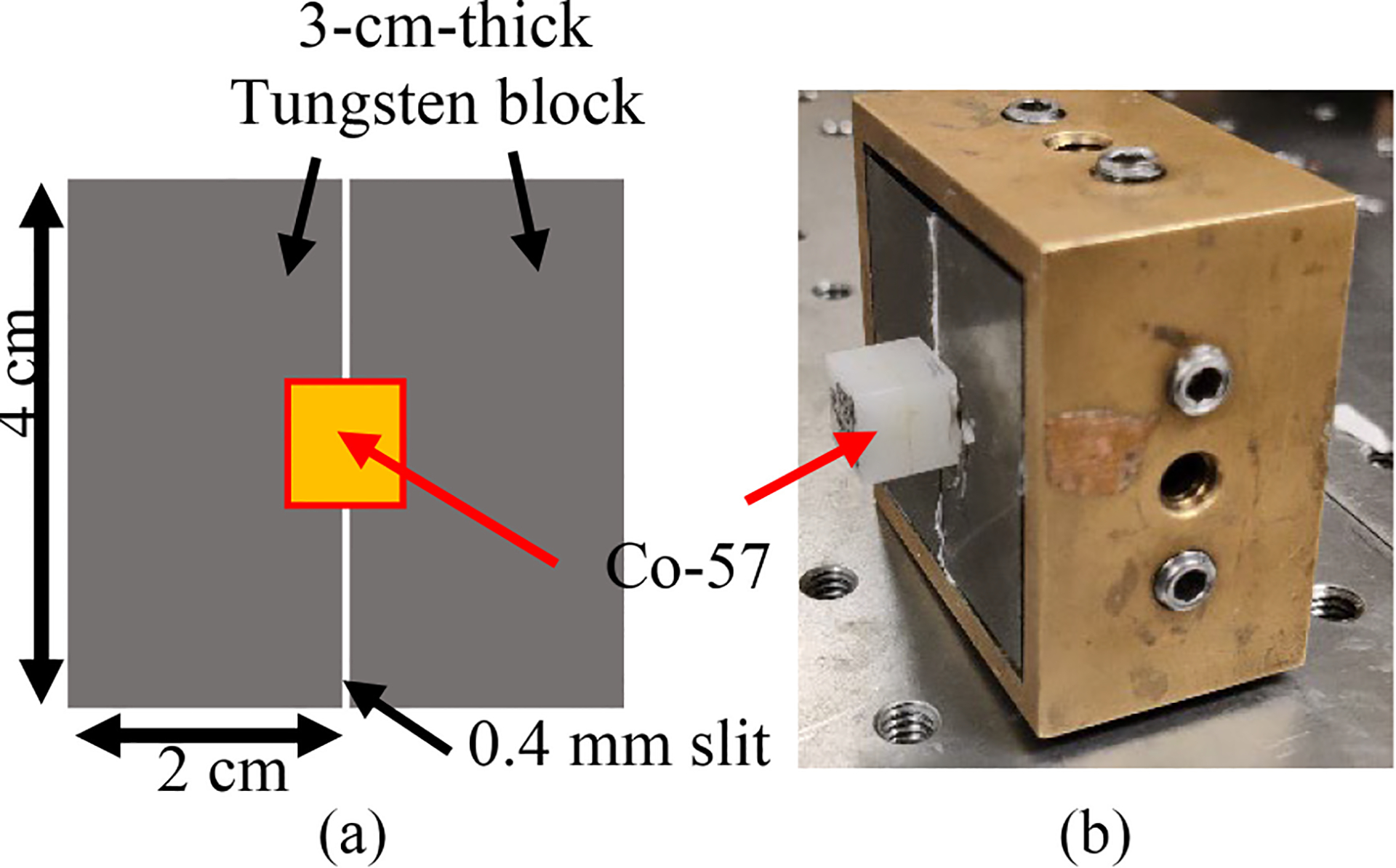
(a) Schematic of the Co-57 sheet beam. (b) Experimental setup of the sheet beam.

The performance of large volume CZT imaging detectors often suffers from spatial distortion and nonuniformity in detection efficiency across the active volume. Fig. 2 shows two experimental projections synthesized from a Co-57 sheet beam scanning, illustrating the significant spatial distortion in x - and y-directions. The sheet beam is produced with a tungsten slit collimator and a Co-57 point source as shown in Fig. 3. The Co-57 point source is ~50μCi and 0.25 mm in diameter in the center of the cube. Considering that the distance between the collimator and the front surface of the detector is 1 cm, the actual beam size irradiating the detector is ~0.5 mm due to geometrical divergence of the sheet beam.

### B. Maximum-Likelihood-Based Data Preconditioning

For the CZT detector used in this study, we acknowledged its accuracy of DOI information and divided it into 5 DOI layers with the first layer at the anode side (the detector’s rear). Without loss of generality, we define the raw projection $\hat{f}$ as the counts of gamma-ray interactions recorded at mth DOI layer of raw detector-pixels, which includes all the artifacts and distortions. The proposed data preconditioning technique is to recover f from $\hat{f}$ through

(1) $$f={𝒫}_{E}(\hat{f})=\text{arg}\;\underset{f}{\text{max}}[L(\hat{f}|f)]$$

where f is the preconditioned projection cancelling the artifacts and distortion. f is with regards to the preconditioned detector-pixels at the same DOI layer. 𝒫 denotes the operator of preconditioning for a given energy E at which the projection is acquired.

Based on the assumption of Poisson-distributed photon counts, the probability of observing $\hat{f_{j}}$ counts in the jth raw detector-pixel given an expected count $\lambda_{j}$ is

(2) $$p\left({\hat{f}}_{j}|\lambda_{j}\right)=\frac{\lambda^{{\hat{f}}_{j}}e^{-\lambda_{j}}}{{\hat{f}}_{j}!}$$

where $\lambda_{j}=\sum_{i}P_{ji}f_{i}.P_{ji}$ is the probability that a gamma-ray interacting in the preconditioned detector-pixel i is located in raw pixel j by the subpixel algorithm. Therefore, the likelihood function is expressed as

(3) $$L(\hat{f}|f)=\prod_{j}p\left({\hat{f}}_{j}|\lambda_{j}\right)$$

and the log-likelihood function is

(4) $$\text{ln}\left(L\left(\hat{f}|f\right)\right)=\sum_{j}\left[{\hat{f}}_{j}\;\text{ln}\left(\lambda_{j}\right)-\lambda_{j}-\text{ln}\left({\hat{f}}_{j}!\right)\right].$$

This equation can be solved with the maximum-likelihood expectation-maximization (MLEM) algorithm [32] to find the estimated projection maximizing the likelihood function, which is the preconditioned projection f. The MLEM algorithm is an iterative process. In this study, the update rule is

(5) $$f_{l}^{\left(t+1\right)}=\frac{f_{l}^{\left(t\right)}}{s_{l}}\sum_{j=1}^{M}\frac{P_{\text{jl}}\hat{f_{j}}}{\sum_{i=1}^{N}P_{\text{ji}}f_{i}^{\left(t\right)}}$$

where $s_{l}=\sum_{j=1}^{M}P_{\text{jl}}$ is the sensitivity referring to the probability that a gamma-ray reaching the preconditioned detector-pixel l is detected. In addition, M=440×440 is the number of raw detector-pixels while N is the number of preconditioned detector-pixels. $P_{\text{ji}}$ is the DRF

(6) $$\text{DRF}=\left(\begin{array}{ccc} P_{11} & \cdots & P_{1N}\\ \vdots & P_{ij} & \vdots \\ P_{M1} & \cdots & P_{\text{MN}} \end{array}\right)$$

which needs to be modelled experimentally. In this DRF matrix, the jth row represents the probability distribution on the preconditioned detector plane given a gamma-ray detected by the raw detector-pixel j. The lth column is the raw detector response to the gamma-ray originated from the preconditioned detector-pixel l. Note that this discussion considers only one DOI layer and a specific energy, although technically, 5 DRFs should exist for all layers at any given energy.

It is worth noting that the MLEM algorithm effectively redistributes the measured counts and preserves the total number of counts in the projection.

### C. Modeling of Detector Response

The ideal way to obtain the DRF is to experimentally scan across the detector with a pencil beam. If the pencil beam irradiates the detector-pixel j, the measured projection $I_{j}$ on the detector scaled by the total number of emitted gamma-rays is the jth column of DRF. However, this approach is impractical for our detectors due to the significant time required, especially considering the need to obtain DRFs for five different DOI layers and the extensive number of pixels and the large number of detectors involved.

**Fig. 4.**
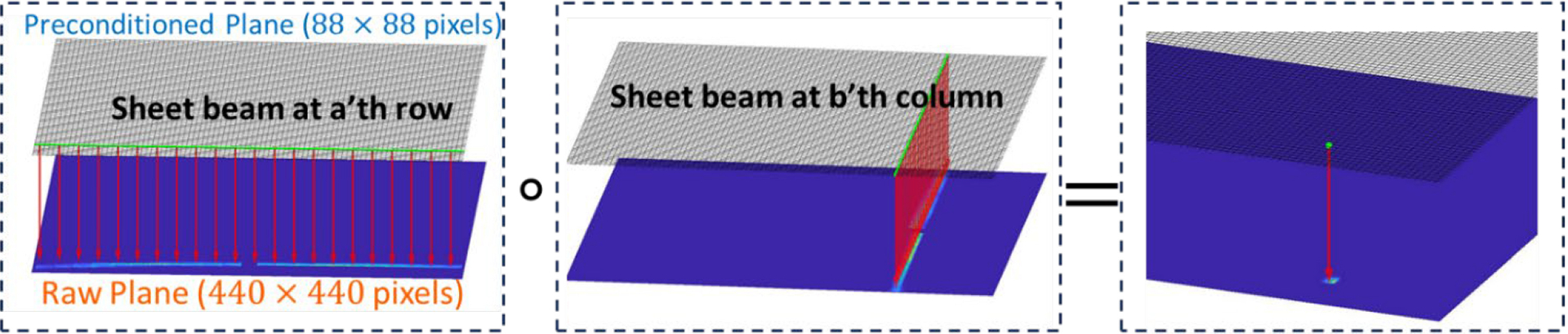
Illustration of obtaining the approximate detector response to a pencil beam with two corresponding sheet beams. The top grids denote the preconditioned plane which defines the position of pencil or sheet beam. The bottom plane denotes the raw projection to the beams (detector response).

In this study, we used a sheet beam scanning approach to obtain the approximate DRF [23]. Suppose the preconditioned detector-pixel j is on the ath row and the bth column. To obtain the jth column of DRF which is the raw detector response to the gamma-ray originated from the preconditioned detector-pixel j, we used a sheet beam to irradiate the ath row and the bth column, respectively, of the predefined preconditioned detector plane. Given considerations for scanning time, the preconditioned detector plane is set to N=88×88 with a pixel pitch of 0.5 mm to balance the spatial resolution and scanning efficiency. The raw detector projections to the sheet beams are denoted as $R_{a}$ w.r.t. the ath row or $C_{b}$ w.r.t the bth column. The approximate projection ${\tilde{I}}_{j}$ is computed as

(7) $${\tilde{I}}_{j}≅R_{a}\circ C_{b}$$

where ∘ denotes the element-wise multiplication as illustrated in Fig. 4. Then, the DRF is obtained approximately by

(8) $$\text{DRF}≅\left[{\tilde{I}}_{1},{\tilde{I}}_{2},\ldots ,{\tilde{I}}_{N}\right].$$

The sensitivity is calculated based on the approximate DRF. To summarize, for one detector, we need 88 scanning positions per direction with an interval of 0.5 mm (this interval should match the pitch of preconditioned detector-pixels), and in turn, 88 × 2 scanning positions in total. The sheet beam is of 0.5 mm width and made up of a Co-57 point source and slit collimator as illustrated in Fig. 3. An energy window of [120 keV, 124 keV], which included > 90% of photopeak events and can effectively reduce scattering events, was used to obtain the projections. The nonuniform intensity distribution of the sheet beam, attributed to the point source, introduces nonuniformity that necessitates correction.

While the approximation effectively corrects spatial distortions, it introduces blurring of high-resolution details, making it inadequate for precisely addressing the abnormal pixel boundary issue at a scale of approximately 0.2 mm. Moreover, the spatial distortion patterns in detectors used in the DE-SPECT are found to be consistent across various DOI layers and energies. Consequently, a single DRF can be employed to manage spatial distortions across all DOI layers and energies. We could use 𝒫 to denote the data preconditioning for all DOI layers and energies in the following sections. Note that values in the approximate DRF are not scaled or normalized, which can be large due to the multiplication of projections. To achieve accurate counts in the preconditioned projections, a calibration factor is introduced during the uniformity correction process, allowing for straightforward adjustment to correct for nonuniformity and ensure the integrity of quantitative measurements.

### D. Pre- and Post-Processing

**Fig. 5.**
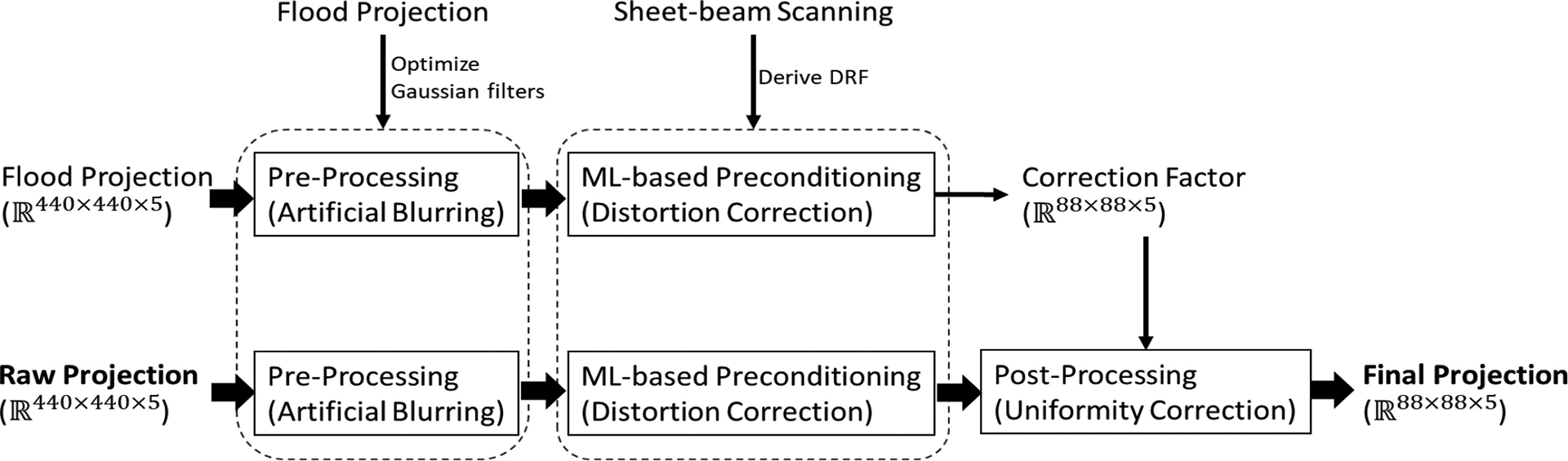
Whole framework of maximum-likelihood-based data preconditioning technique.

Due to the loss of high-resolution information and the nonuniform intensity in sheet beam, extra pre- and post-processing are required to deal with the abnormal pixel boundary issue (Fig. 1) and the nonuniformity issue to a flood field. In this study, we implement artificial blurring as preprocessing and uniformity correction as post-processing. The whole framework is illustrated as Fig. 5.

For the artificial blurring, we first conducted a flood measurement. Since the pattern of pixel boundary issue is similar with regards to different anode pixels, we took one anode pixel and subdivide it into different regions to apply different 2-D Gaussian filters. Based on the projection within this anode pixel, we used MATLAB to optimize the parameters of Gaussian filters to minimize the variation of counts in these regions. Note that within an anode pixel, the projections at different depths are not the same. Therefore, they need to be optimized independently. Given a raw projection $A_{m}$ at layer m, the preprocessing is to smooth it to ${𝒮}_{m}\left(A_{m}\right)$ with the optimized Gaussian filters.

The uniformity correction for the detector is performed based on the flood measurements. This correction effectively addresses any nonuniformity that may have arisen earlier, whether from the nonuniform sheet beam or the crystal itself. With the prior information of the attenuation coefficient of the detector and the flood source, the flood measurement should lead to a flat projection and the counts at different layers, c(m) should follow:

(9) $$c(m)=c_{0}\int_{(n-m)t}^{(n-m+1)t}e^{-\mu_{\text{total}}(E)\cdot x}\mu_{\text{PE}}(E)\text{dx}$$

where n=5 is the number of DOI layers and t=2mm denotes the thickness of each layer. $\mu_{\text{total}}(E)$ and $\mu_{\text{PE}}(E)$ are the total and photoelectric linear attenuation coefficient of CZT at energy E, respectively. $c_{0}$ is the number of events at the front surface of the detector. Therefore, theoretically, the ratio of c(m) to c(n) should obey

(10) $$\text{ratio}\left(m\right)=\frac{c\left(m\right)}{c\left(n\right)}=e^{-\mu_{\text{total}}\left(E\right)\cdot \left(n-m\right)t}-e^{-\mu \cdot \left(n-m+1\right)t}.$$

However, due to the nonuniformity of the crystal as well as the nonuniform profile of the sheet beam, the projection from the flood source is not flat and the ratio does not follow the theory. In the uniformity correction, given the raw flood projection FP, we compute the correction factor of the layer $m,CF_{m}\in R^{88\times 88}$ by

(11) $${\text{CF}}_{m}=\frac{\text{ratio}\left(m\right)}{\text{ratio}\left(n\right)}⊘\;𝒫\left({𝒮}_{m}\left({\text{FP}}_{m}\right)\right)\cdot \frac{\sum {\text{FP}}_{n}}{\sum_{l=1}^{N}\text{sgn}\left(s_{l}\right)}$$

where $FP_{m}$ denotes the projection at layer $m,\sum FP_{n}$ calculates the total number of events in the raw projection at layer n. ⊘ denotes the element-wise division and sgn is the sign function. In this correction, we calibrate the counts at each layer based on the attenuation by setting the counts at layer n (closest to the cathode) as the reference. Overall, given a raw projection A, the final projection $B_{m}$ at layer m is calculated by

(12) $$B_{m}=𝒫\left({𝒮}_{m}\left(A_{m}\right)\right)\circ CF_{m}.$$

While the distortion patterns observed at different energies are similar, allowing the use of a single DRF for distortion correction across all energies within our framework, the flood projections differ significantly. Consequently, it is necessary to develop distinct blurring filters and corresponding correction factors tailored for each energy level to ensure accurate image correction and uniformity across the system.

### E. Phantom Study

**Fig. 6.**
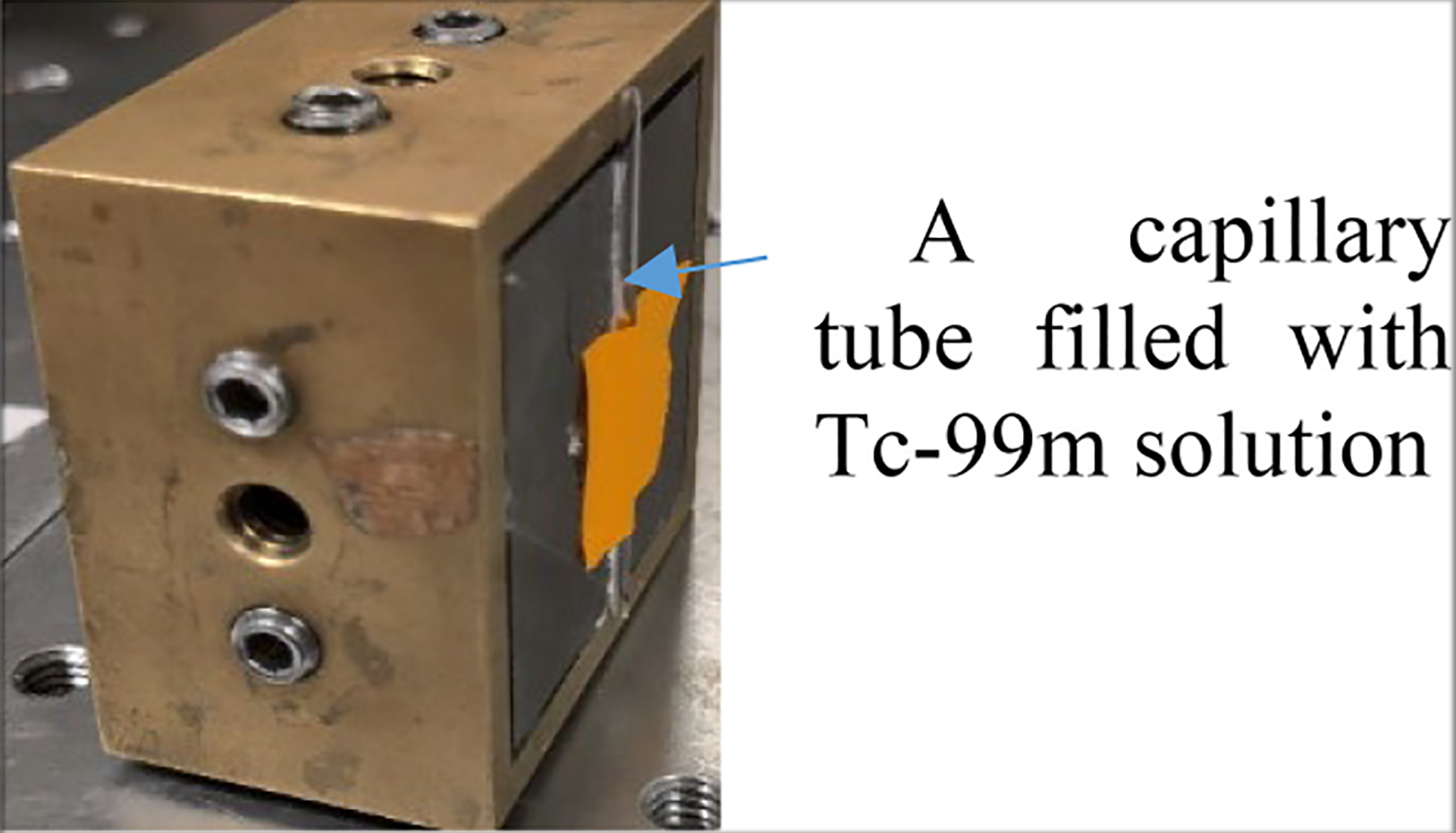
Experimental setup of the Tc-99 m sheet beam.

We carried out preliminary studies with Tc-99m to validate the effectiveness of the proposed technique in both projection and image domain. An energy window of [136 keV, 144 keV] was used to generate projections. We created a 140 keV sheet beam using a slit collimator and a capillary tube filled with Tc-99m shown in Fig. 6. The thickness and width of the slit are 3 cm and 0.5 mm, respectively. The distance between the slit and the detector is 1.5 cm. Considering the geometrical divergence, the actual beam size irradiating the detector is about 0.5 mm × [(3+1.5)mm/3 mm] = 0.75 mm. Employing this beam, we systematically scanned across the detector with an interval of 4.5 mm, allowing for an assessment of impact of preconditioning on spatial resolution and distortion in the projection domain. Spatial resolution was evaluated by applying Gaussian fitting to the preconditioned projections to determine the FWHM of the peaks. To assess spatial distortion, we calculated the discrepancies between the fitted centroids and the actual scanned positions, and using root-mean-square-errors (RMSEs) to provide a quantitative measure of accuracy in the projection domain.

**Fig. 7.**
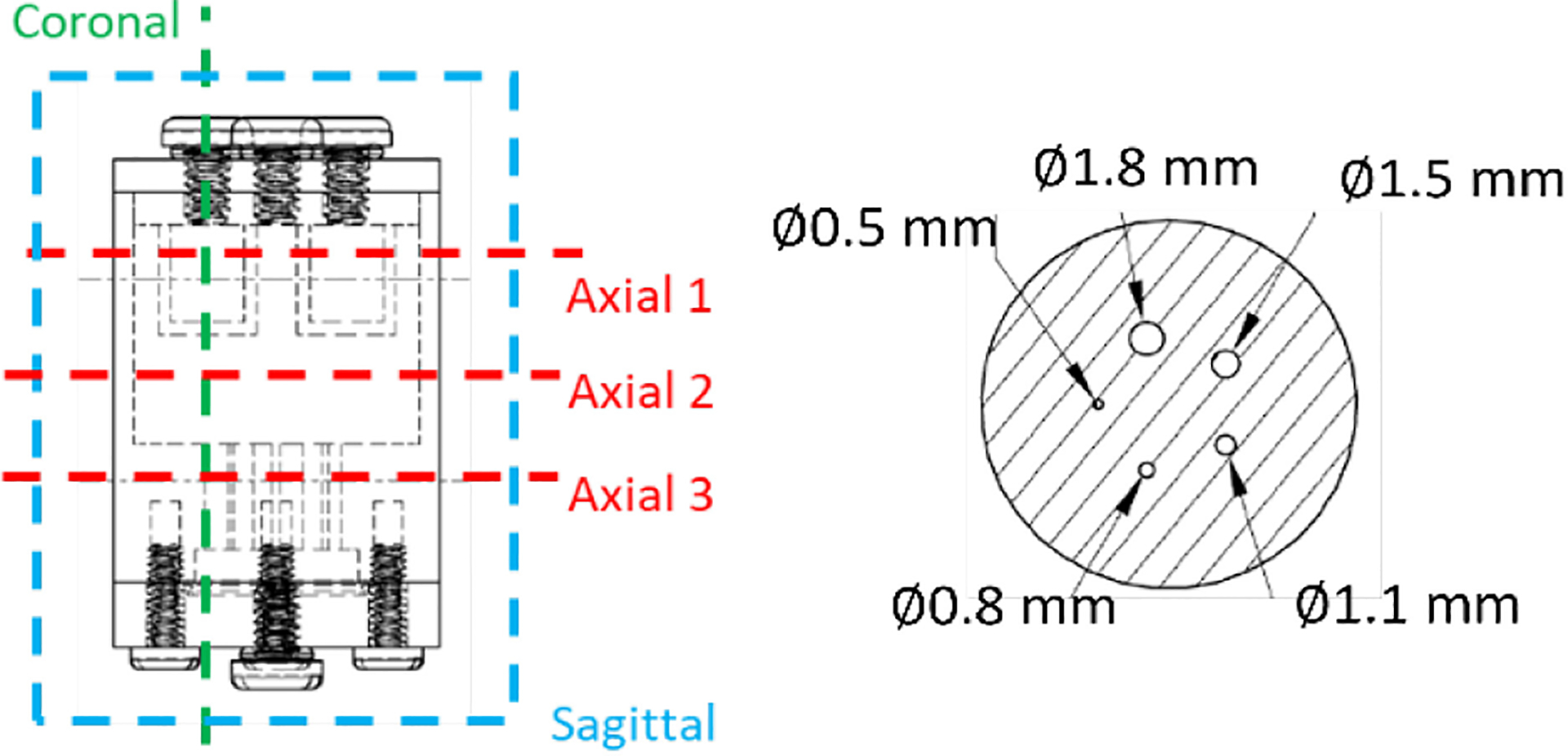
Schematic of the IQ phantom [33] and 5 defined views in reconstruction. (The image is used with permission).

We also conducted an imaging study with National Electrical Manufacturers Association (NEMA) standard IQ phantom [33] from Phantech Medical as shown in Fig. 7. For this imaging study, we built a simple prototype with a 1 mm D pinhole and a CZT module (2.2 × 2.2 × 1.0 cm^3^). The distances of detector-to-pinhole and pinhole-to-object are the same (9 cm). In this setup, the field-of-view is projected to the CZT module through the pinhole. To ensure enough angular sampling, we used a high-resolution rotation stage (Model: SR50PP, Newport) to hold the IQ phantom and 24 angles were rotated with an interval of 15°. The phantom was filled with 1 mCi Tc-99m solution. The acquisition time was 10 min for each angle and the decay of Tc-99m was compensated in acquired projections. To quantify the results from the phantom imaging, we calculated the normalized standard deviations within uniform areas and the Peak-to-Valley (P/V) ratios. For the P/V ratio, a line profile was drawn through the centers of two air holes, identifying two valleys and three peaks along the line. The P/V ratios were then calculated as the ratio of the height of the lower peak to the height of the valley, which provides a measure of the contrast resolution capability of the imaging system.

### F. Experimental Calibration of System Geometry

In this study, we used experimentally acquired projections to derive the geometrical parameters of the prototype system. This method is briefly described below.

For the CZT crystal in the detector module, we first defined a local coordinate system within a global coordinate system and used 6 parameters to characterize the origin and orientation of the local system within the global system. In addition, we used 3 parameters to define the position of the pinhole, 4 parameters to describe the orientation of the sample rotation stage and rotation radius, and 3 parameters to describe the direction of the vertical linear translation stage. This geometrical definition leads to a total of 19 parameters to define the prototype setup, which is denoted as the vector α.

To evaluate these geometrical parameters, we used a Co-57 point-source with a 0.25 mm core diameter mounted on the rotation stage and positioned at roughly 6 mm away from the rotation axis. We rotated the source at 8 uniformly spaced angles across 360°, and then translated the rotation stage along the rotation axis in three axial positions to acquire a total of 24 projections. An energy window of [120 keV, 124 keV] was used to obtain the projections. The weighting centers of these projections were used for the process of calibration, which are denoted as $W_{c}$. By considering the pinhole as a single point and knowing the translation distances and rotation angles as prior information, we can derive the conditional probability, $p\left(W_{c}|\alpha \right)$. The most probable system parameters, $\hat{\alpha }$, can be found by a constrained minimization process

(13) $$\hat{\alpha }=\text{argmin}\left\{-p\left(W_{c}|\alpha \right)\right\}$$

which is performed in MATLAB [34] using the FMINCON function.

In this study, we aimed to compare the performance of the preconditioned projections and raw projections. It is important to note that the preconditioned detector plane is determined exclusively by the scanning process, which may introduce slight displacement and rotation relative to the raw detector plane. Consequently, the system parameters for raw and preconditioned (final) projections differ, necessitating separate calibrations for each set of projections before their respective application in the reconstruction process.

### G. Image Reconstruction

Using pixelated projections (w/ and w/o data preconditioning) from 24 angles, we reconstructed the images with the MLEM algorithm [32]. The update equation is as follows:

(14) $$Q_{l}^{\left(t+1\right)}=\frac{Q_{l}^{\left(t\right)}}{g_{l}}\sum_{j=1}^{n}\frac{a_{\text{jl}}D_{j}}{\sum_{i=1}^{m}a_{\text{ji}}Q_{i}^{\left(t\right)}}$$

where Q is the source distribution, $Q_{i}^{\left(t\right)}$ is the tth iteration of the activity at source-voxel i. The sensitivity $g_{l}$ is the probability that a gamma-photon originated from source-voxel l is detected. $D_{j}$ is the intensity of detector-voxel j in the projection. $a_{\text{jl}}$ is the probability of a photon originating from source-voxel l being detected at detector-voxel j, so-called system response function (SRF). In this equation, n is the number of detector-voxels, including 5 DOI layers of preconditioned detector-pixels while m is the number of source-voxels. To calculate SRF, we utilized a voxel-driven method [35] based on the calibrated geometry, taking into account both the attenuation and the DOI response of the detector.

## III. Results

### A. Results of DRF Modelling

**Fig. 8.**
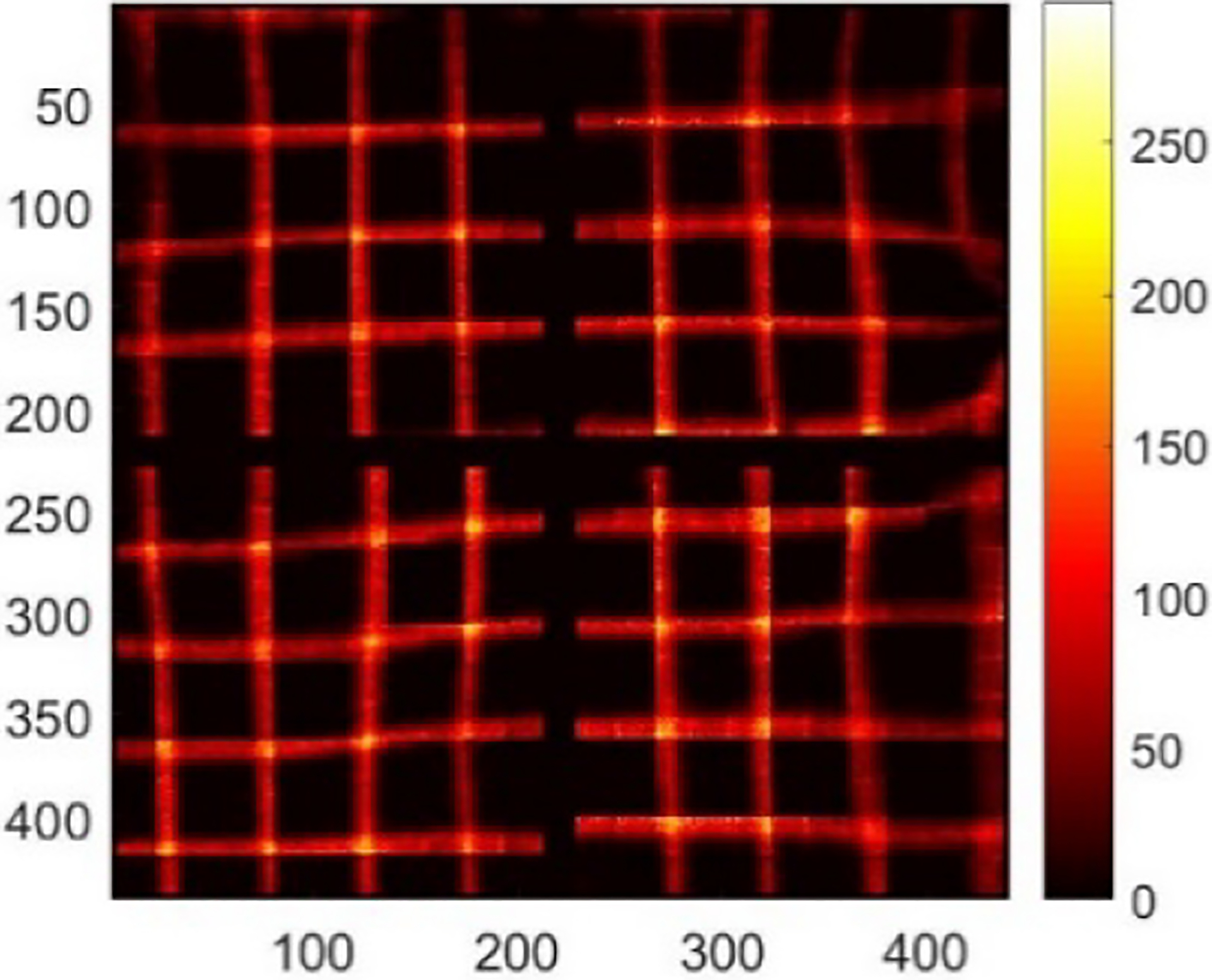
Synthesized raw projections acquired in 2-D Co-57 sheet beam scanning. The step size is 5 mm.

**Fig. 9.**
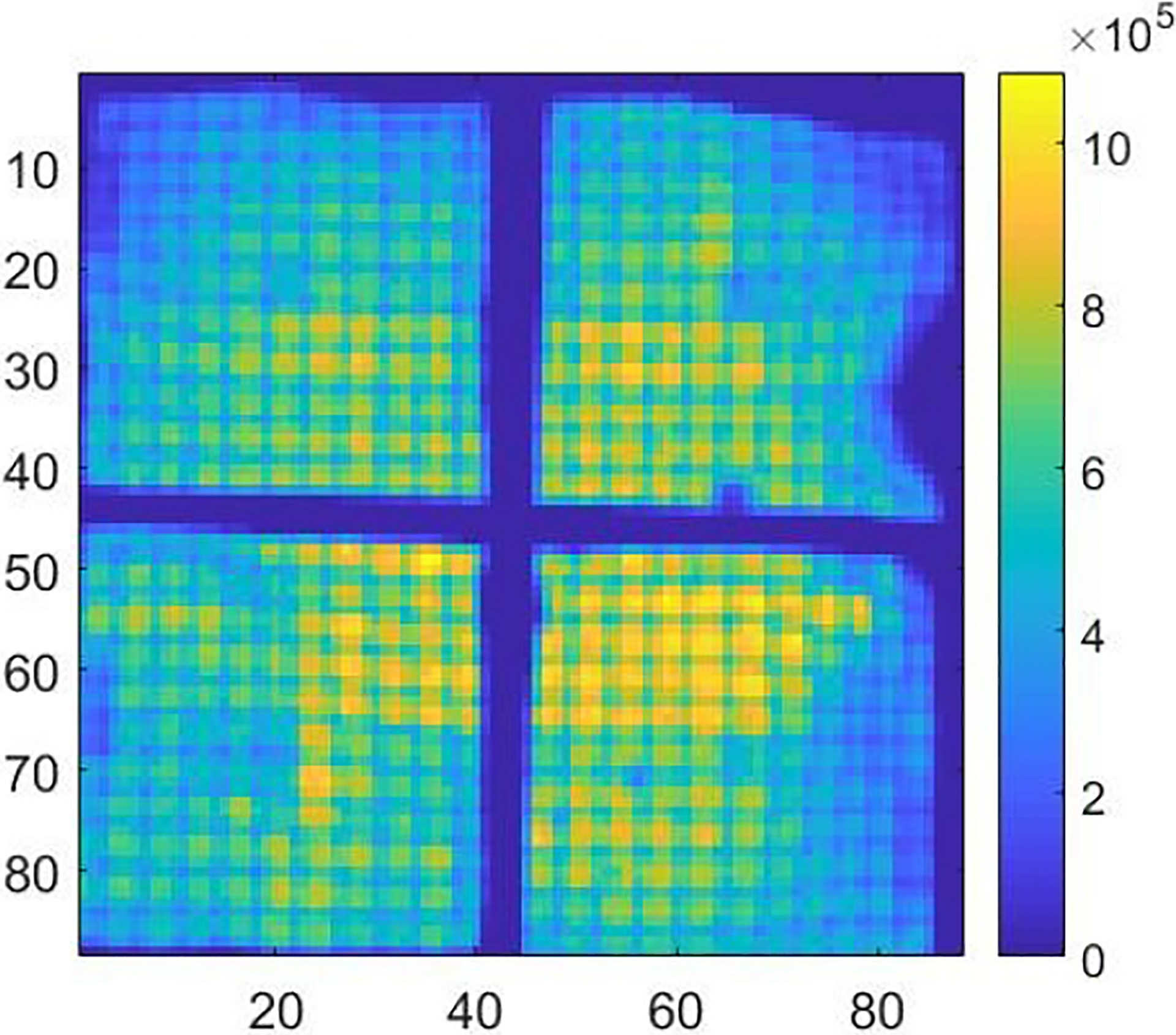
Sensitivity map obtained in 2-D sheet beam scanning.

Among 48 detectors in the DE-SPECT system, we selected the detector with the serial number of M-00-01-043 for the demonstration in this study. From the Co-57 sheet beam scanning, we synthesized the raw projections with the interval of 5 mm in two directions as shown in Fig. 8 combining all depths. Note that Fig. 8 shows a different distortion pattern from Fig. 2, which highlights the fact that the distortion could be different from unit to unit. With the scanning data, we derived the DRF as described in Section II-C. Fig. 9 shows the sensitivity map which refers to $s_{l}=\sum_{j=1}^{M}P_{\text{jl}}$ in Section II-B.

**Fig. 10.**
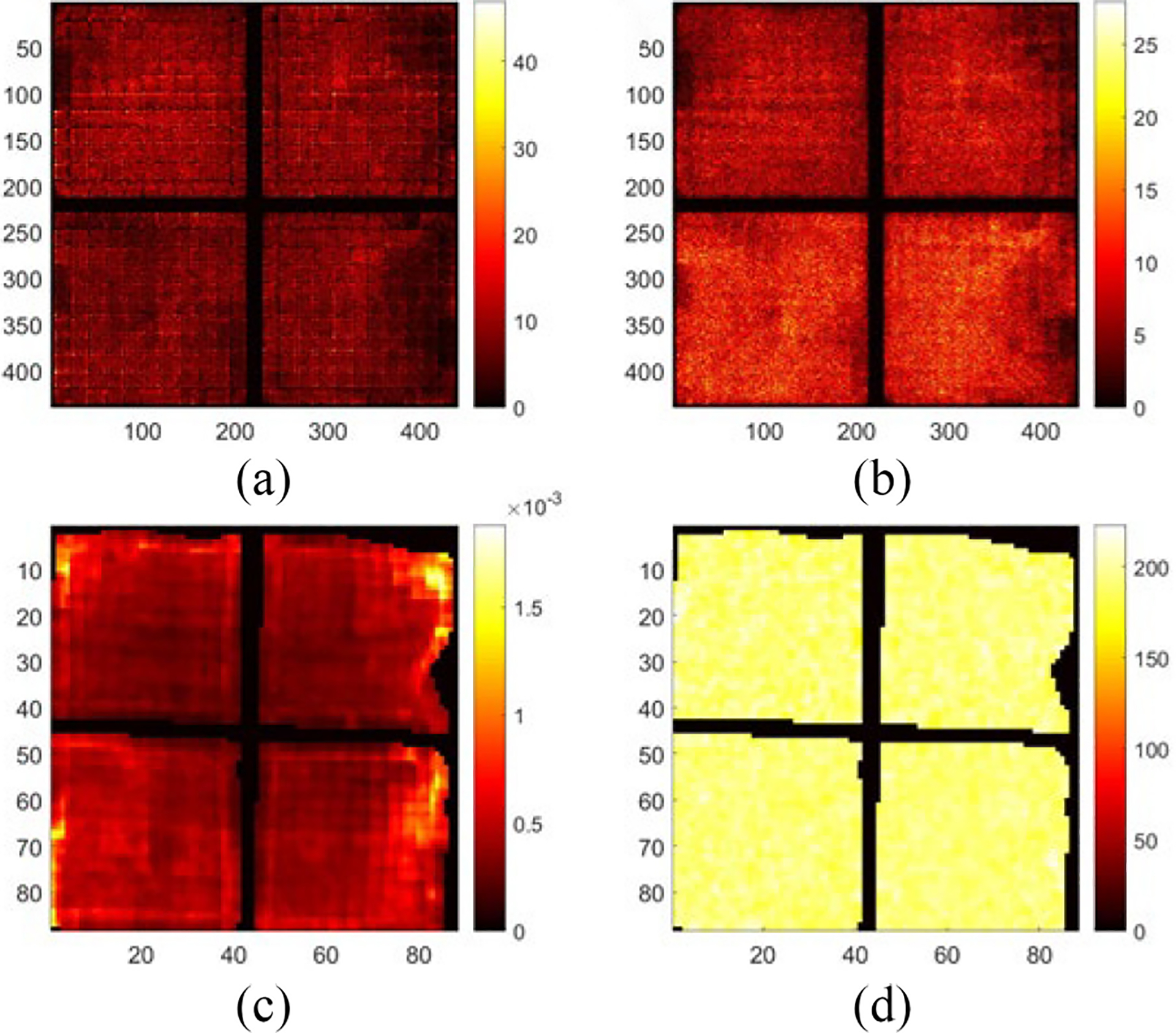
Projections acquired from a Co-57 flood measurement. (a) Raw projection. (b) Projection after artificial blurring (preprocessing). (c) Projection after distortion correction (reconstructed with DRF). (d) Final projection after uniformity correction (post-processing).

**Fig. 11.**
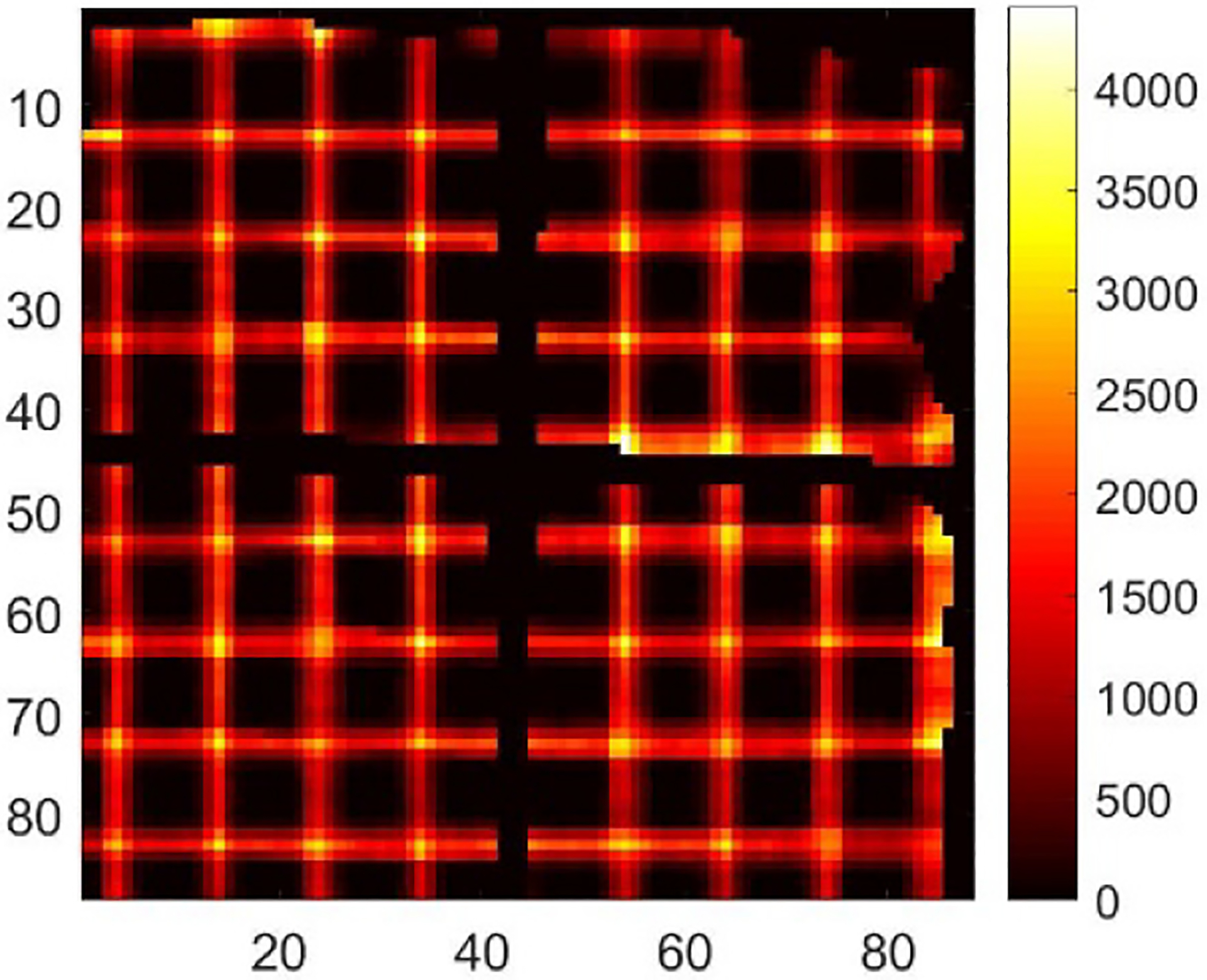
Final projection given out by the whole preconditioning framework with the input of the synthesized raw scanning projections (Fig. 8).

Fig. 10(a) shows the raw projection at DOI layer 5 from a Co-57 flood measurement, ${FP}_{5}$. Fig. 10(b) shows the projection, ${𝒮}_{m}\left(FP_{m}\right)$, after preprocessing. Fig. 10(c) shows the projection after maximum-likelihood-based preconditioning, $𝒫\left({𝒮}_{m}\left(FP_{m}\right)\right)$. Fig. 10(d) shows the final projection, $B_{5}$ after post-processing. Fig. 11 shows the final projection in comparison to the synthesized scanning projection (Fig. 8). Since they are also used to generate the DRF, the corrected lines are perfectly horizontal or vertical.

### B. Comparison of Projections W/O and W/Preconditioning

**Fig. 12.**
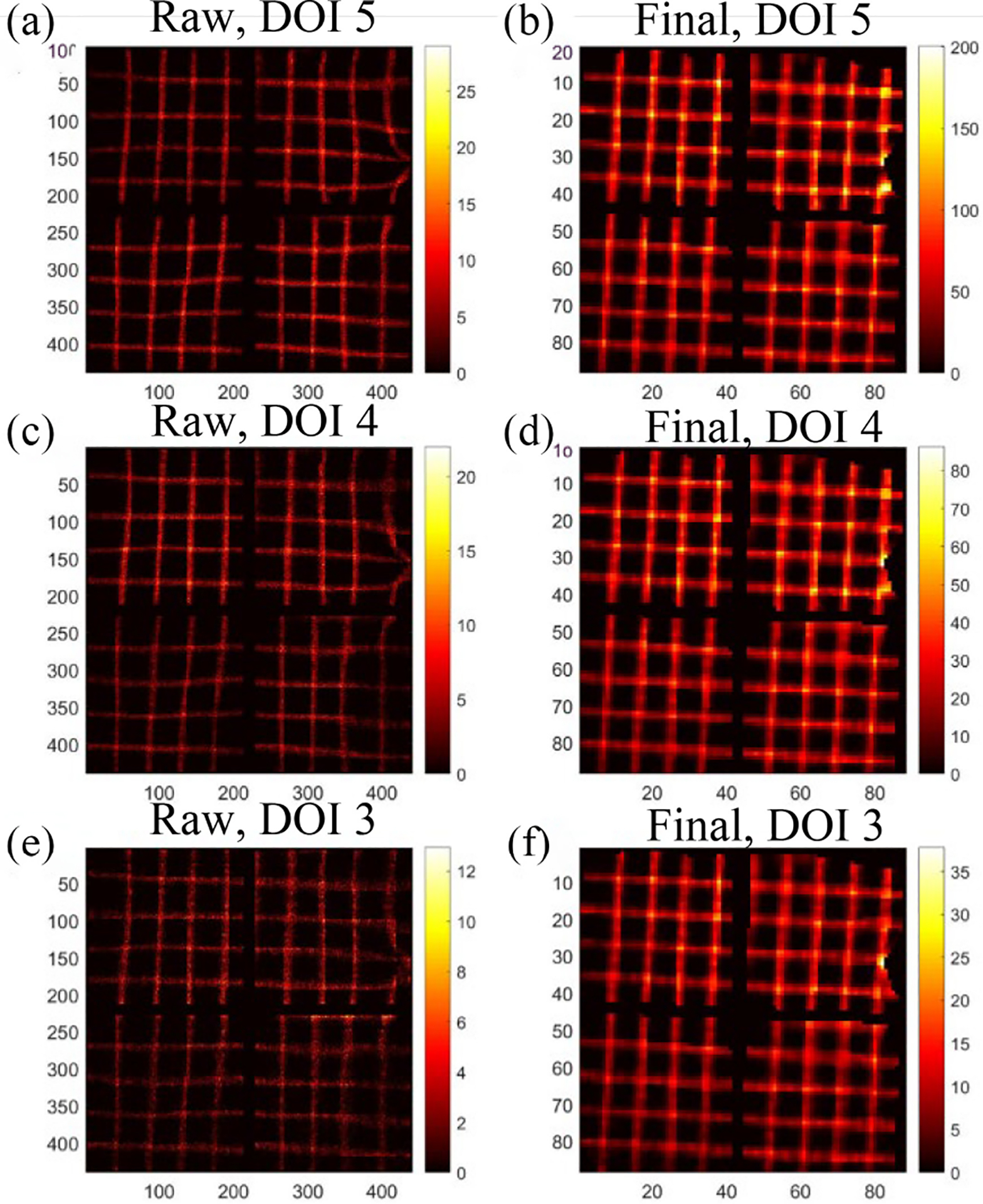
Raw and final projections synthesized from Tc-99m sheet beam scanning. (a) Raw projection at DOI layer 5. (b) Final projection at DOI layer 5. (c) Raw projection at DOI layer 4. (d) Final projection at DOI layer 4. (e) Raw projection at DOI layer 3. (f) Final projection at DOI layer 3.

As described in Section II-E, the results of Tc-99m sheet beam scanning are as shown in Fig. 12. The projections at DOI layers 3~5 are shown while we ignore layer 1 and layer 2 because they have few counts. Fig. 12(a), (c), and (e) shows the raw projections acquired at DOI layer 5 to 3. Fig. 12(b), (d), and (f) shows the final projections with regards to Fig. 12(a), (c), and (e), respectively. The final projections in Fig. 12 (second column) are straight at different depths, illustrating the consistent distortion patterns at different DOIs. In addition, according to the attenuation coefficient, the counts in Fig. 12(c) should be theoretically ~0.4 of the counts shown in Fig. 12(a). This discrepancy illustrates the nonuniformity across different depths, which is also corrected in final projections.

However, compared to Fig. 11, the final projections in Fig. 12 are not vertical or horizontal. This is because the orientation of the Tc-99m sheet beam was slightly tilted and different from the Co-57 sheet beam used scanning to obtain the DRF.

**Fig. 13.**
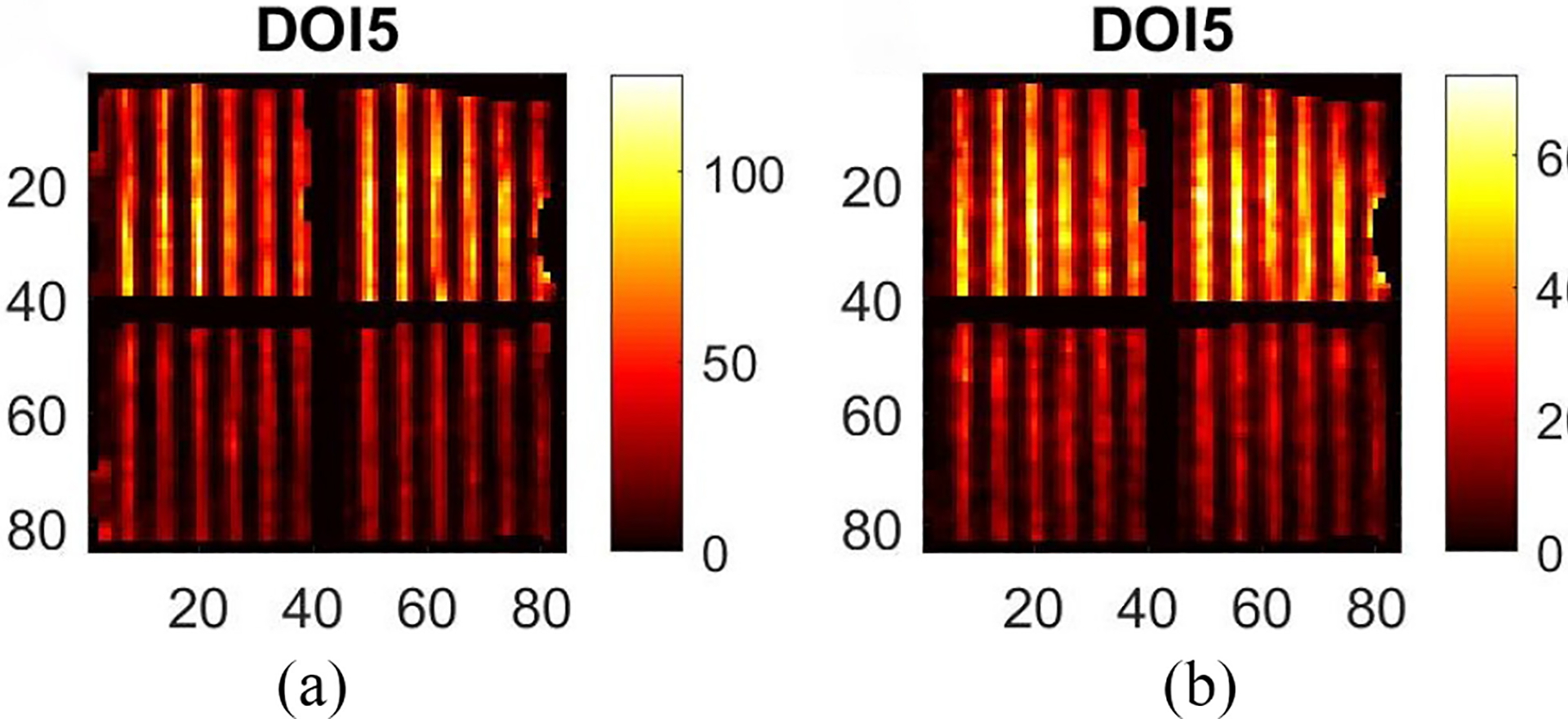
Final projections synthesized from In-111 sheet beam scanning. (a) Final projection DOI layer 5 at 171 keV. (b) Final projection at DOI layer 5 at 245 keV.

Fig. 13 depicts final projections synthesized from In-111 sheet beam scanning by the same slit collimator as for Tc-99m. These projections were preconditioned by the DRF obtained from Co-57 sheet beam scanning, illustrating the consistent distortion patterns at different energies as mentioned Section II-C.

**Fig. 14.**
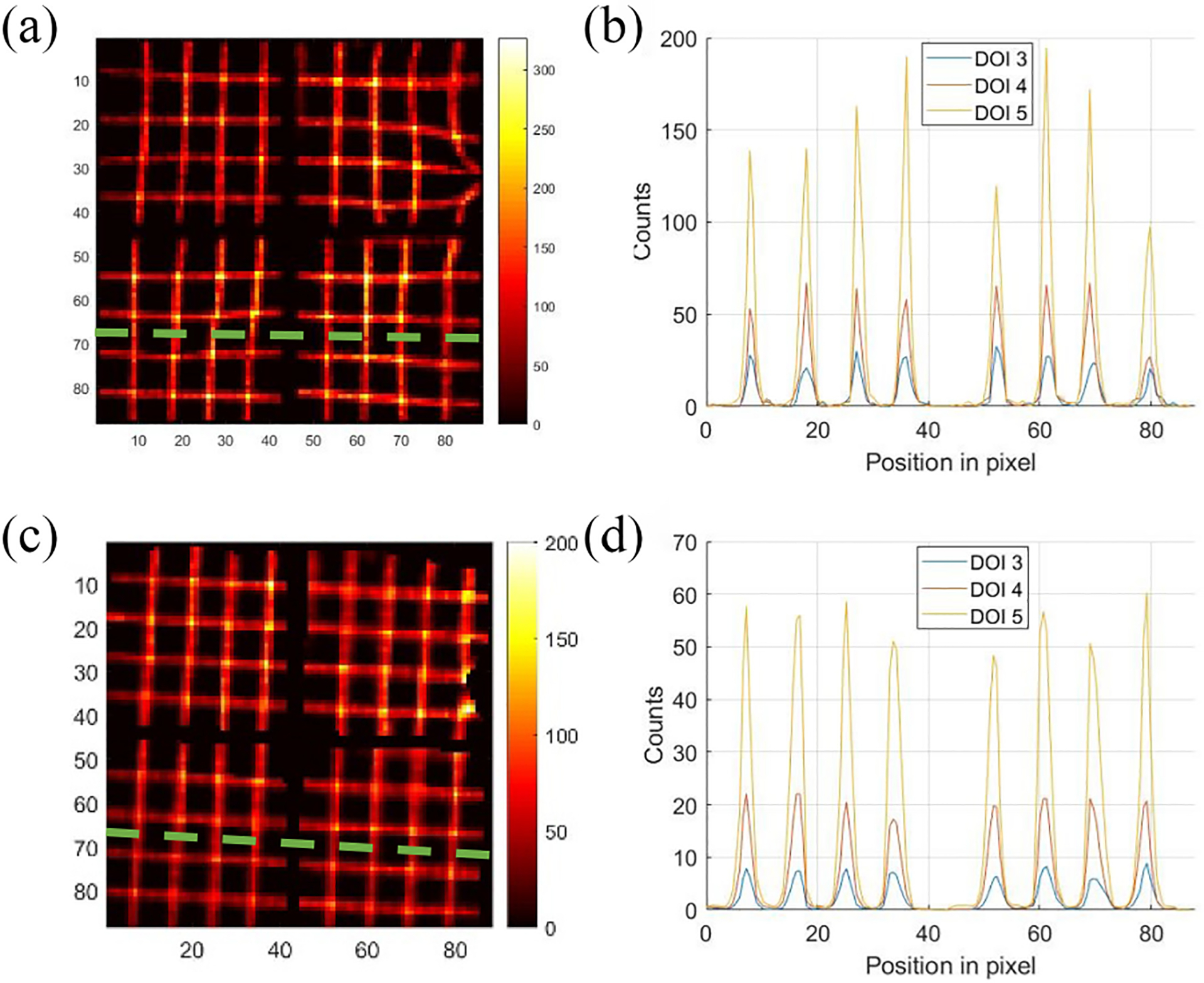
(a) Raw projection (DOI 5) synthesized from the Tc-99m sheet beam scanning. (b) Line profiles going through the dashed line in (a) at DOI layer 3~5. (c) Final projection (DOI 5) preconditioned from (a). (d) Line profiles going through the dashed line in (c) at DOI layer 3~5.

**Fig. 15.**
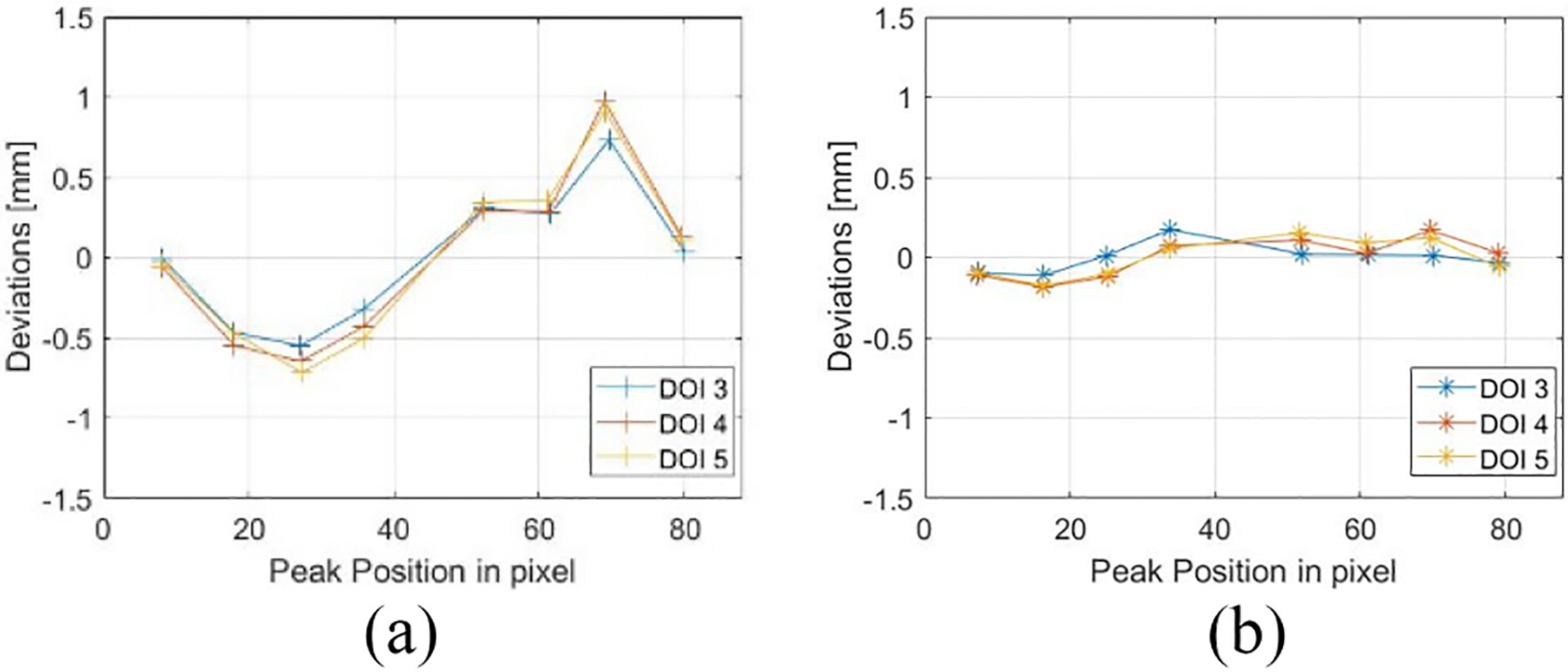
Discrepancies between the fitted centroids and the actual scanned positions. (a) Raw projection. (b) Preconditioned projection.

The cross sections through the dashed lines at different depths with and without preconditioning are shown in Fig. 14. Fig. 14(a) and (b) displays the line profiles of the raw projection, illustrating the nonuniformity for different depths. Note that we rebinned the raw projection from 440 × 440 to 88 × 88 for more counts. Fig. 14(c) and (d) depicts the line profiles of the final projection. Gaussian fitting of the curve in DOI layer 5 helped determine peak positions and calculate the FWHMs, which averaged 0.9 mm for raw and 1.1 mm for final profiles. Given that the beam width reaching the detector is around 0.75 mm, the estimated intrinsic spatial resolutions of the detector before after data preconditioning are approximately $\sqrt{0.9^{2}-0.75^{2}}=0.5\;\text{mm}$ and $\sqrt{1.1^{2}-0.75^{2}}=0.8\;\text{mm}$ FWHM, respectively. This estimated raw intrinsic spatial resolution matched the previously reported value, while the final resolution becomes worse due to the implementation of the artificial blurring in preprocessing. The alteration also made the peaks in Fig. 14(b) sharp and high while the peaks in Fig. 14(d) are wider and lower. With the line profiles shown in Fig. 14(b) and (d), we calculated the discrepancies between the fitted centroids and the actual scanned positions for these eight peaks. The results for different DOI layers before and after preconditioning are shown in Fig. 15. The RMSEs at different DOI depths for the raw and final line profiles are ~0.54 and ~0.12 mm, respectively, demonstrating the accuracy of the proposed preconditioning technique.

### C. Comparison of Reconstruction W/O and W/ Data Preconditioning

In the process of geometrical calibration, we aimed to individually calibrate the geometrical parameters using both raw and final projections. However, the calibration with raw projections failed to converge to a reasonable result, primarily due to significant distortion. Recognizing that the differences in geometrical parameters between raw and final data are limited to the position and orientation of the detector, we adopted the other geometrical parameters, describing the rotation, and the pinhole position, from the calibration with the final data. This approach allowed us to successfully complete the calibration for the raw data by incorporating the calibrated geometrical parameters from the final projections.

**Fig. 16.**
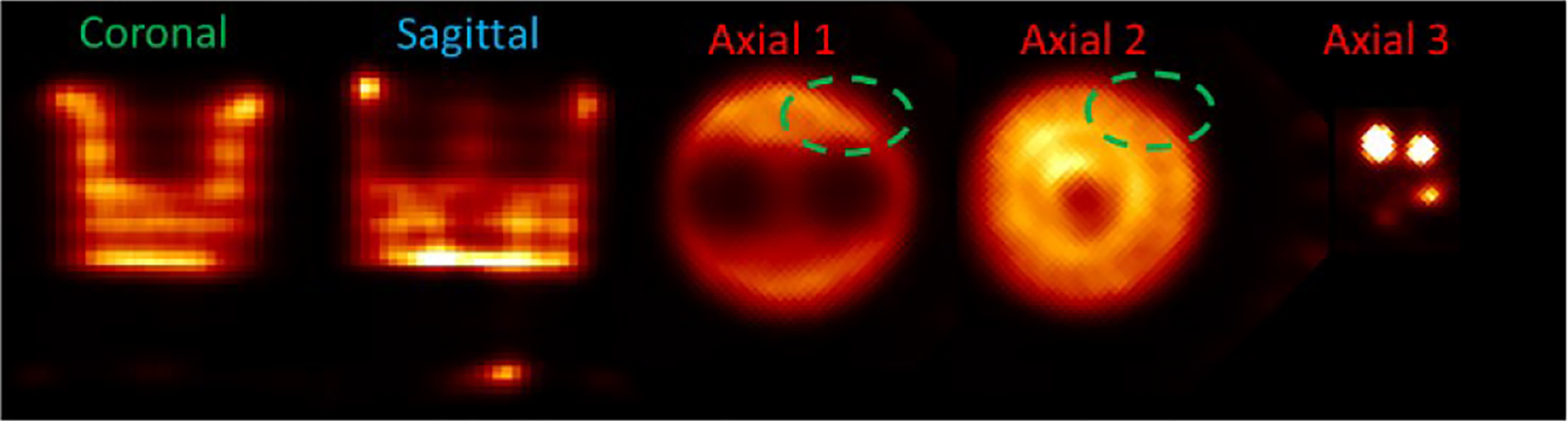
Reconstructed image (10th iteration) of the Tc-99m IQ phantom with raw data and geometrical calibration. The 5 views are axial sections of the phantom defined in Fig. 8. Some obvious deformations are highlighted with dashed circles.

**Fig. 17.**
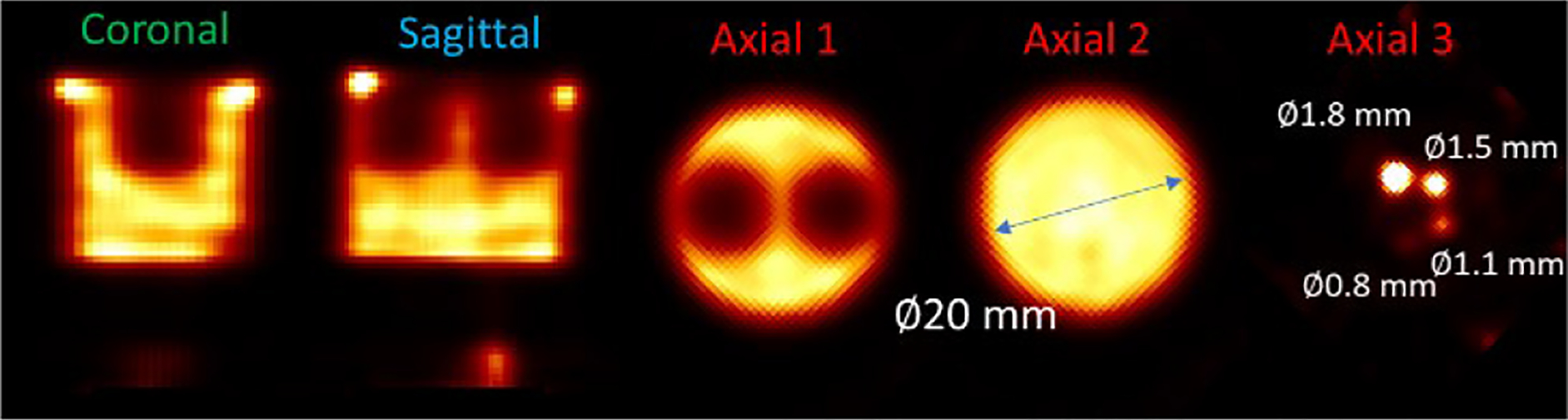
Reconstructed image (15th iteration) of the Tc-99m IQ phantom with preconditioned data and geometrical calibration.

In the IQ phantom study, we acquired ~1.7 million gamma rays inside the [136 keV, 144 keV] energy window. The images reconstructed using the MLEM algorithms and with raw projections and final projections are shown in Figs. 16 and 17, respectively. Since we used MLEM to reconstruct the images, to select iteration numbers of images for different energy resolutions, we control the fitted FWHMs of the 1.8 mm rods to be consistent. In the reconstruction, the source space contains 64 × 64 × 64 voxels with the size of 0.5 mm × 0.5 mm × 0.5 mm. Five axial sections of the phantom, defined in Fig. 7, are presented for analysis. In the image reconstructed with raw projections, periodic features attributable to pixel boundaries in the detector are evident (Fig. 16), whereas these artifacts are significantly mitigated in the image reconstructed with data preconditioning (Fig. 17). Notably, the raw projection image exhibits some deformation, as highlighted by the dashed circles, distorting the expected round shapes. Conversely, the image processed with data preconditioning displays no such deformation (Fig. 17)

**Fig. 18.**
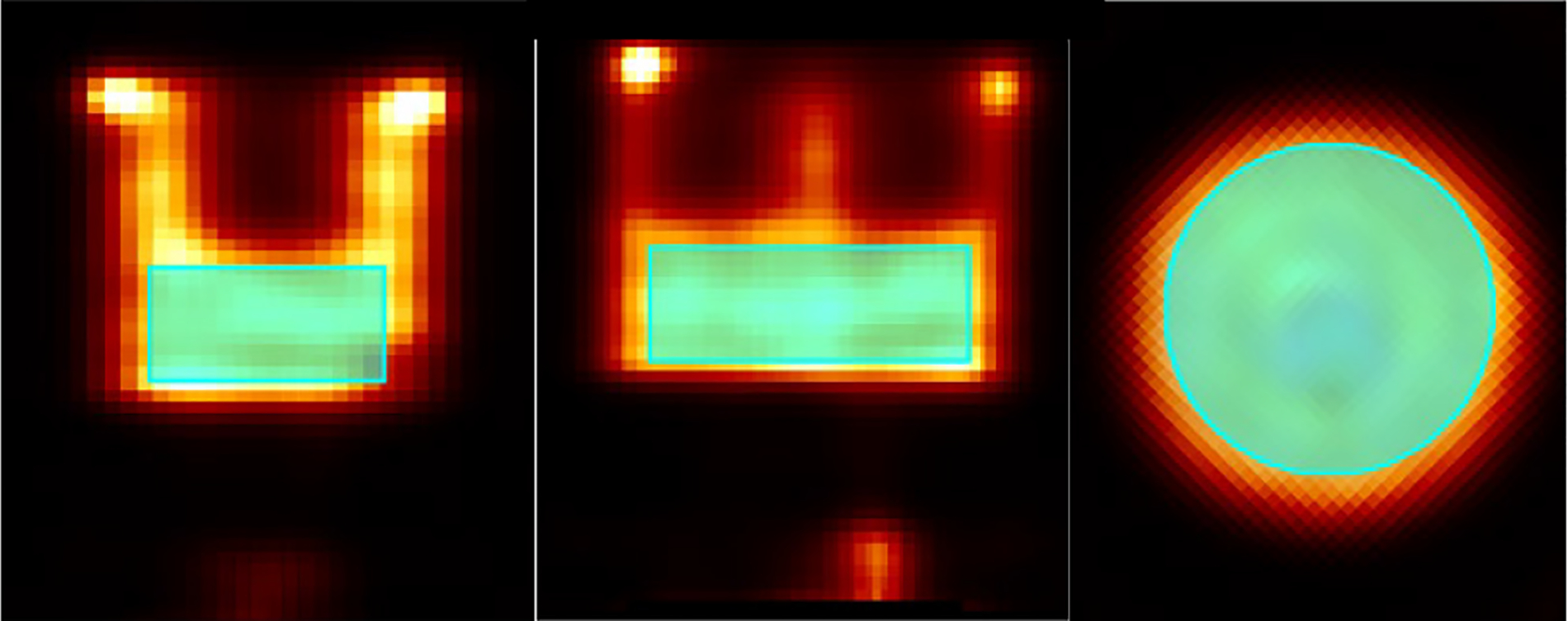
Uniform area selected for calculating the normalized standard deviations is highlighted by cyan.

**Fig. 19.**
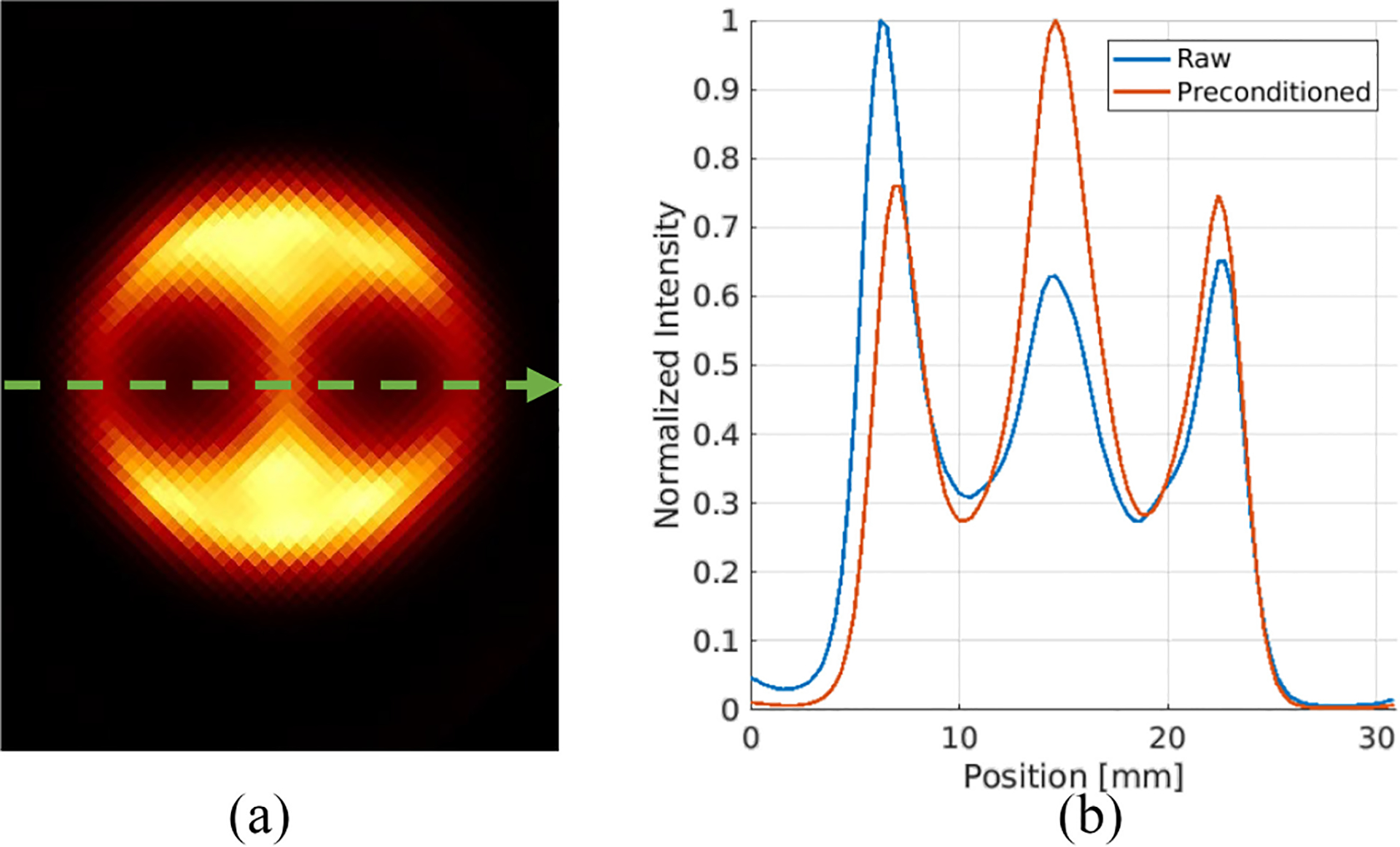
(a) Axial view 1 of the preconditioned image. (b) Normalized line profiles of raw and preconditioned images going through the dashed arrow shown in (a).

To further analyze the imaging results, we selected a uniform area of 770 mm^3^ as shown in Fig. 18. The normalized standard deviations within this area for raw and preconditioned images were measured at 0.21 and 0.06, respectively. In addition, line profiles through the specified dashed arrow in Fig. 19(a) for both image types were plotted in Fig. 19(b). According to Section II-E, the Peak-to-Valley (P/V) ratios for the two valleys in the raw image were 2.03 and 2.36, while in the preconditioned image, they improved to 2.76 and 2.68. These metrics highlight the enhanced uniformity and contrast in the preconditioned images.

## IV. Discussion

In this study, we address a critical issue for using 3-D position-sensitive CZT detectors used for SPECT imaging applications. Although these detectors offer superior energy and spatial resolutions, they present three major issues: 1) pixel boundary issues; 2) spatial distortions; and 3) nonuniformity, which limit their immediate applicability in imaging. Our proposed maximum-likelihood-based data preconditioning technique, while primarily developed for CZT detectors, has broader implications. We have demonstrated the effectiveness of the proposed technique with a CZT detector in both projection and imaging domains. Comparatively, the spatial distortion and nonuniformity issues we tackle are not exclusive to CZT detectors but are prevalent in various imaging sensors. Our proposed technique only utilizes final spectral and spatial information for correction. This makes it applicable to other position-sensitive detectors as well. This adaptability underscores the potential universality of our approach in the field of medical imaging.

The successful application of our technique relies on the construction of the DRF for each detector. The most straightforward and accurate method is to use pencil beam scanning with the time complexity of O(N), where N is the number of preconditioned detector-pixels. This process is extremely time-consuming. We utilized an alternative way of using sheet beam to perform 2-D scanning, effectively reducing the time complexity to $O(\sqrt{N})$. However, this method yields an approximate DRF, that is insufficient for addressing the abnormal pixel boundary issue which contains high-frequency details. With regards to the pixel boundary issue, we applied an artificial blurring to smooth out the square-grid pattern in the raw detected event map. We did not describe the details about the artificial blurring in this paper because the pixel boundary issue is uniquely created by its subpixel positioning.

Note that while distortion patterns across different DOIs and energies are similar within the detectors utilized in this study, the high-resolution details vary. Theoretically, as mentioned in Section II-B, different DRFs corresponding to various DOIs and energies would be necessary. However, as detailed in Section II-C, our method of using sheet beam scanning to obtain an approximate DRF blurs these details. This approximation allows us to use a single DRF, derived from Co-57 sheet beam scanning to effectively correct spatial distortions across all DOI layers and energies, which was also illustrated in Figs. 12 and 13.

In addition to spatial distortion and pixel boundary issue, the detectors suffer from nonuniformity issue similarly to other detectors. For detectors used in this study, the nonuniformity issue combines several aspects: 1) the nonuniformity of crystal (similar to other detectors) and 2) nonuniformity introduced by scanning. *First*, the issue caused by the nonuniformity of crystal could be potentially corrected by the precise DRF as described in Section II-B. However, for the sake of practical use, a single DRF is used in this study which cannot correct the nonuniformity in depth direction. *Second*, in our sheet beam scanning, we used a Co-57 point source to produce the sheet beam. This makes the intensity distribution of the sheet beam nonuniform and hard to calibrate. Hence, there exists the nonuniformity issue in estimating sensitivity from DRF, which in turn, leads to the nonuniformity in the resultant projection as well. Note that values in the approximate DRF are not scaled or normalized, which can be large due to the multiplication of projections. A factor needs to be multiplied to the preconditioned projections reconstructed with this DRF to get correct number of counts. Therefore, we took a post uniformity correction to address the above problem. For detectors without DOI capabilities, uniformity correction redistributes counts in the x- and y-directions without altering the total counts. However, for our detectors with DOI capabilities, direct application of scaling factors to achieve uniform counts in three dimensions is incorrect due to nonuniformity in depth and attenuation. We utilized ratios derived from (9) and (10) to adjust total counts for each DOI layer, using the layer closest to the front surface, with records the most events as a baseline. While these ratios aim to align with the ground truth, exact matches are not guaranteed. For precise quantitative SPECT imaging, further calibration of counts is necessary.

We have demonstrated quantitatively that spatial distortion is effectively addressed in the projection domain with the discrepancies between the measured and actual peak positions and the RMSEs improved from 0.54 to 0.12. However, the reconstruction process tends to obscure these distortions, making their impact less apparent in the image domain and more challenging to quantify. Despite these complexities, we still rely on normalized standard deviations and P/V ratios to showcase the enhancements brought by our preconditioning technique.

However, this technique has several limitations. A primary limitation lies in the time constraints associated with 2-D scanning. Our current methodology utilizes a single slit for beam generation. The scanning remains time-consuming despite the use of a strong source. A promising solution could be the adoption of a multislit collimator to generate multiple sheet beams simultaneously, potentially reducing scanning time significantly. This approach would require advanced post-processing for effective projection segmentation to extract single line projections. In this study, a single DRF was employed for all DOI layers because it is time consuming to acquire sufficient counts for deeper layers due to the attenuation of CZT for 122 keV. This approach, while efficient, could introduce parallax error if the sheet beam is not perpendicular to the detector plane. Ideally, employing multiple DRFs tailored to different DOI layers would solve this issue. A potential solution to achieve adequate counts for deeper layers involves using higher energy beams, inspired by findings that spatial distortion remains consistent across different energy levels. Another challenge involves the inherent constraints of 2-D sheet beam scanning in accurately capturing high-resolution information. The obtained DRF is only an approximation. For a more precise DRF, exploring the use of a parallel-hole collimator to generate multiple pencil beams could be beneficial, albeit accompanied by the need of multiple point sources or high-concentration fluid source, and the same need of projection segmentation. In addition, the preprocessing method used in our study mitigates but does not completely eliminate pixel boundary issues. As a result, periodic features are still visible in the final images. Furthermore, the implementation of artificial blurring degrades the intrinsic spatial resolution from ~0.5 to 0.8 mm.

Future research should focus on developing faster, more accurate scanning techniques. The potential of our technique to be universally applicable across various types of detectors requires further verification in future research. Such advancements could significantly improve the quality and diagnostic accuracy of medical imaging, benefiting a wide range of medical and research applications.

## V. Conclusion

In this study, we have introduced a novel maximum likelihood-based data preconditioning technique for 3-D position-sensitive CZT detectors, effectively addressing the pivotal challenges in the DE-SPECT system. Our approach has successfully resolved issues related to pixel boundaries, spatial distortions, and nonuniformity, thereby significantly enhancing precision of locating gamma-ray interaction and resultant projections and images. The implications of our technique extend beyond CZT detectors, suggesting broader applications in various imaging sensors. Looking ahead, the potential for this technique to be adapted to other types of imaging sensors presents an exciting opportunity for advancements in the field of medical imaging. This research represents a substantial leap forward, contributing to the development of more accurate, efficient, and versatile imaging technologies and ultimately laying the groundwork for improved diagnostic methodologies and patient care.
